# Supplementary material for: Comparative effectiveness of adjunct non-pharmacological interventions on maternal and neonatal outcomes in gestational diabetes mellitus patients: A systematic review and network meta-analysis protocol of randomized controlled trials
Source: PLoS One. 2022 Jan 27;17(1):e0263336. doi: 10.1371/journal.pone.0263336 (PMC8794170; doi:10.1371/journal.pone.0263336)
Supplement: S1 Table — Proposed search strategy in PubMed and its relevance. (DOCX) [file pone.0263336.s001.docx]

# S1 Table. Proposed search strategy in PubMed and its relevance

| **Search date** | **Search terms & their combination** | **No. Of results** | **Electronic link** | **Pre-identified citations retrieved** | | |
| --- | --- | --- | --- | --- | --- | --- |
|  |  |  |  | **DOI** | **Citation position on relevancy wise sorting*** | **Intervention type** |
| 09-Jam-2022 | (("diabetes, gestational"[MeSH Terms] OR "gestational diabetes mellitus"[Title/Abstract] OR "GDM"[Title/Abstract]) AND "randomized controlled trial"[Publication Type]) AND (randomizedcontrolledtrial[Filter]) | 691 | [Link](https://pubmed.ncbi.nlm.nih.gov/?term=%28%22diabetes%2C+gestational%22%5BMeSH+Terms%5D+OR+%22gestational+diabetes+mellitus%22%5BTitle%2FAbstract%5D+OR+%22GDM%22%5BTitle%2FAbstract%5D%29+AND+%28randomizedcontrolledtrial%5BFilter%5D%29&filter=pubt.randomizedcontrolledtrial&size=200) | DOI: 10.1186/s12884-019-2258-y | 16 | Nutritional intervention |
|  |  |  |  | DOI: 10.1016/j.wombi.2017.10.004 | 15 | Exercise intervention |
|  |  |  |  | DOI: 10.1136/bmjopen-2019-030884 | 251 | Digital intervention |
| 09-Jam-2022 | (("nutrition*"[All Fields] OR ("vitamin s"[All Fields] OR "vitamine"[All Fields] OR "vitamines"[All Fields] OR "vitamins"[Pharmacological Action] OR "vitamins"[MeSH Terms] OR "vitamins"[All Fields] OR "vitamin"[All Fields]) OR "probitic"[All Fields] OR ("synbiotics"[MeSH Terms] OR "synbiotics"[All Fields] OR "synbiotic"[All Fields]) OR ("prebiotically"[All Fields] OR "prebiotics"[MeSH Terms] OR "prebiotics"[All Fields] OR "prebiotic"[All Fields]) OR ("calcification, physiologic"[MeSH Terms] OR ("calcification"[All Fields] AND "physiologic"[All Fields]) OR "physiologic calcification"[All Fields] OR "mineralization"[All Fields] OR "mineral s"[All Fields] OR "mineralisable"[All Fields] OR "mineralisation"[All Fields] OR "mineralisations"[All Fields] OR "mineralise"[All Fields] OR "mineralised"[All Fields] OR "mineralising"[All Fields] OR "mineralizations"[All Fields] OR "mineralize"[All Fields] OR "mineralized"[All Fields] OR "mineralizer"[All Fields] OR "mineralizers"[All Fields] OR "mineralizes"[All Fields] OR "mineralizing"[All Fields] OR "minerals"[MeSH Terms] OR "minerals"[All Fields] OR "mineral"[All Fields]) OR ("digital"[All Fields] OR "digitalisation"[All Fields] OR "digitalised"[All Fields] OR "digitalization"[All Fields] OR "digitalize"[All Fields] OR "digitalized"[All Fields] OR "digitalizer"[All Fields] OR "digitalizing"[All Fields] OR "digitally"[All Fields] OR "digitals"[All Fields] OR "digitization"[All Fields] OR "digitizations"[All Fields] OR "digitize"[All Fields] OR "digitized"[All Fields] OR "digitizer"[All Fields] OR "digitizers"[All Fields] OR "digitizes"[All Fields] OR "digitizing"[All Fields]) OR ("smartphone"[MeSH Terms] OR "smartphone"[All Fields] OR "smartphones"[All Fields] OR "smartphone s"[All Fields]) OR ("exercise"[MeSH Terms] OR "exercise"[All Fields] OR "exercises"[All Fields] OR "exercise therapy"[MeSH Terms] OR ("exercise"[All Fields] AND "therapy"[All Fields]) OR "exercise therapy"[All Fields] OR "exercise s"[All Fields] OR "exercised"[All Fields] OR "exerciser"[All Fields] OR "exercisers"[All Fields] OR "exercising"[All Fields]) OR ("educability"[All Fields] OR "educable"[All Fields] OR "educates"[All Fields] OR "education"[MeSH Subheading] OR "education"[All Fields] OR "educational status"[MeSH Terms] OR ("educational"[All Fields] AND "status"[All Fields]) OR "educational status"[All Fields] OR "education"[MeSH Terms] OR "education s"[All Fields] OR "educational"[All Fields] OR "educative"[All Fields] OR "educator"[All Fields] OR "educator s"[All Fields] OR "educators"[All Fields] OR "teaching"[MeSH Terms] OR "teaching"[All Fields] OR "educate"[All Fields] OR "educated"[All Fields] OR "educating"[All Fields] OR "educations"[All Fields]) OR ("counsel"[All Fields] OR "counseled"[All Fields] OR "counselings"[All Fields] OR "counselled"[All Fields] OR "counselling"[All Fields] OR "counseling"[MeSH Terms] OR "counseling"[All Fields] OR "counsellings"[All Fields] OR "counsels"[All Fields])) AND (("diabetes, gestational"[MeSH Terms] OR "gestational diabetes mellitus"[Title/Abstract] OR "GDM"[Title/Abstract]) AND "randomized controlled trial"[Publication Type] AND "randomized controlled trial"[Publication Type])) AND (randomizedcontrolledtrial[Filter]) | 399 | [Link](https://pubmed.ncbi.nlm.nih.gov/?term=%28nutrition%2A+OR+vitamin+OR+probitic+OR+synbiotic+OR+prebiotic+OR+mineral+OR+digital+OR+smartphone+OR++exercise+OR+education+OR+counseling%29+AND+%28%28%22diabetes%2C+gestational%22%5BMeSH+Terms%5D+OR+%22gestational+diabetes+mellitus%22%5BTitle%2FAbstract%5D+OR+%22GDM%22%5BTitle%2FAbstract%5D%29+AND+%28randomizedcontrolledtrial%5BFilter%5D%29+AND+%28randomizedcontrolledtrial%5BFilter%5D%29%29&sort=date&filter=pubt.randomizedcontrolledtrial&size=200&ac=no) | DOI: 10.1186/s12884-019-2258-y | 10 | Nutritional intervention |
|  |  |  |  | DOI: 10.1016/j.wombi.2017.10.004 | 15 | Exercise intervention |
|  |  |  |  | DOI: 10.1136/bmjopen-2019-030884 | 85 | Digital intervention |

*This rank might vary on replication as newly indexed studies in PubMed will populate in the search output
