# Supplementary material for: Comparative effectiveness of adjunct non-pharmacological interventions on maternal and neonatal outcomes in gestational diabetes mellitus patients: A systematic review and network meta-analysis protocol of randomized controlled trials
Source: PLoS One. 2022 Jan 27;17(1):e0263336. doi: 10.1371/journal.pone.0263336 (PMC8794170; doi:10.1371/journal.pone.0263336)
Supplement: S2 File — (PDF) [file pone.0263336.s003.pdf]

Proposed data abstraction form

Intervention review of randomized controlled trials

Title: Comparative effectiveness of adjunct non-pharmacological interventions on maternal and neonatal outcomes in gestational diabetes mellitus patients: A systematic review and network meta-analysis protocol of randomized controlled trials.

Use this form to abstract data from each of the studies included in the review.

Author: Dr. Sumanta Saha

* Required

Reviewer author's detail

1. Name *

____

2. Date of data abstraction *

____

Example: January 7, 2019

3. Email id *

____

Is the study population eligible for inclusion in this review?

4. What is diagnosis of trial participants? *

Mark only one oval.

- ◯ Gestational diabetes melitus
- ◯ Other: ______________________

Is the study design eligible for inclusion in this review?

5. What is the design of the study? *

Mark only one oval.

- ◯ Randomized controlled trial
- ◯ Other

Study details

Please enter the details from respective trials

6. Last name of the first author of the publication *

____

7. Year of publication *

____

8. Digital object identifier (if avaialble)

9. PMID (if available)

10. Web link

If a PMID or DOI is not availble for a study, copy the electronic link of the study from your browser and paste it here.

11. Enlist the country or countries where the trial got conducted. *

if conducted in multiple countries, first mention the number of countries and then enlist their names (separated by comma) in the next line.

12. Name the continent where the trial was conducted *

if conducted in multiple countries, first mention the number of countries and then enlist their names (separated by comma) in the next line

Mark only one oval.

- Asia
- Africa
- North America
- South America
- Europe
- More than one continent

13. Trial registration no.

14. The trial is

Mark only one oval.

- Single centered
- Multicentered
- Other:

15. Trial duration (in weeks)

16. Is funding information available in the article?

Mark only one oval.

- ◯ Yes
- ◯ No
- ◯ Other: ____________________

17. Was ethical clearance obtained for conducting the trial?

Mark only one oval.

- ◯ Yes
- ◯ No
- ◯ Other: ____________________

18. Was participant consent obtained from the trial participants?

Mark only one oval.

- ◯ Yes
- ◯ No
- ◯ Other: ____________________

19. Which diagnostic criteria got used to diagnose gestational diabetes mellitus?

Mark only one oval.

- ◯ American Diabetes Association
- ◯ International Association of Diabetes and Pregnancy Study Groups criteria
- ◯ Carpenter and Coustan's criteria
- ◯ European Diabetic Pregnancy Study Group
- ◯ Other: ____________________

20. What was the gestational age of participants (in weeks) when they were recruited in the trial?

Mention in weeks. E.g., if gestational age of participant recruitment in the trial was 24-28 weeks, mention '24-28.'

____

21. Did the study population include women with a previous history of gestational diabetes mellitus?

Mark only one oval.

- ◯ Yes
- ◯ No
- ◯ Unclear
- ◯ Other: ____________________

Type of intervention studied in the trial

22. Choose the type of intervention studied in the trial

If a combination of the following interventions were tested, meniton the those in the 'Other' option

Check all that apply.

- [ ] Nutritional supplementation
- [ ] Digital intervention/s
- [ ] Structured supervised exercise program
- [ ] Structured educational and/or counseling service

Other: [ ] ______________________

Data of nutritional supplement receiving arm/s

Skip this section if the trial didn't test this intervention

23. No. of arms

____

Arm 1

24. Intervention received

____

25. Intervention dosage and regimen

____
____
____
____
____

26. How many participants got randomized?

____

27. No. of males

____

28. Mean age (years, standar deviation)

____

Arm 2

Skip if one treatment arm tested this intervention type.

29. Intervention received

____

30. Intervention dosage and regimen

31. How many participants got randomized?

32. No. of males

33. Mean age (years, standar deviation)

Arm 3

Skip if one treatment arm tested this intervention type.

34. Intervention received

35. Intervention dosage and regimen

36. How many participants got randomized?

37. No. of males

38. Mean age (years, standar deviation)

Arm 4 onwards

Use this section if four or more treatment arms received the intervention type (enter the detail of respective treatment arms in separate lines).

39. Intervention received

40. Intervention dosage and regimen

41. How many participants got randomized?

42. No. of males

43. Mean age (years, standar deviation)

Data of digital intervention receiving arm/s

Skip this section if the trial didn't test this intervention

44. No. of arms

Arm 1

45. Intervention received

46. Intervention dosage and regimen

47. How many participants got randomized?

48. No. of males

49. Mean age (years, standar deviation)

Arm 2

Skip if one treatment arm tested this intervention type.

50. Intervention received

51. Intervention dosage and regimen

52. How many participants got randomized?

53. No. of males

54. Mean age (years, standar deviation)

Arm 3

Skip if one treatment arm tested this intervention type.

55. Intervention received

56. Intervention dosage and regimen

57. How many participants got randomized?

58. No. of males

59. Mean age (years, standar deviation)

Arm 4 onwards

Use this section if four or more treatment arms received the intervention type (enter the detail of respective treatment arms in separate lines).

60. Intervention received

61. Intervention dosage and regimen

62. How many participants got randomized?

63. No. of males

64. Mean age (years, standar deviation)

Data of structured supervised exercise program receiving arm/s

Skip this section if the trial didn't test this intervention

65. No. of arms

Arm 1

66. Intervention received

67. Intervention dosage and regimen

68. How many participants got randomized?

69. No. of males

70. Mean age (years, standar deviation)

Arm 2

Skip if one treatment arm tested this intervention type.

71. Intervention received

72. Intervention dosage and regimen

73. How many participants got randomized?

74. No. of males

75. Mean age (years, standar deviation)

Arm 3

Skip if one treatment arm tested this intervention type.

76. Intervention received

77. Intervention dosage and regimen

78. How many participants got randomized?

79. No. of males

80. Mean age (years, standar deviation)

Arm 4 onwards

Use this section if four or more treatment arms received the intervention type (enter the detail of respective treatment arms in separate lines).

81. Intervention received

82. Intervention dosage and regimen

83. How many participants got randomized?

84. No. of males

85. Mean age (years, standar deviation)

Data of structured educational and/or counseling service receiving arm/s

Skip this section if the trial didn't test this intervention

86. No. of arms

Arm 1

87. Intervention received

88. Intervention dosage and regimen

89. How many participants got randomized?

90. No. of males

91. Mean age (years, standar deviation)

Arm 2

Skip if one treatment arm tested this intervention type.

92. Intervention received

93. Intervention dosage and regimen

94. How many participants got randomized?

95. No. of males

96. Mean age (years, standar deviation)

Arm 3

Skip if one treatment arm tested this intervention type.

97. Intervention received

98. Intervention dosage and regimen

99. How many participants got randomized?

100. No. of males

101. Mean age (years, standar deviation)

Arm 4 onwards

Use this section if four or more treatment arms received the intervention type (enter the detail of respective treatment arms in separate lines).

102. Intervention received

103. Intervention dosage and regimen

104. How many participants got randomized?

105. No. of males

106. Mean age (years, standar deviation)

Data of treatment arm/s receiving a combination of any of the above interventions

Skip this section if the trial didn't test this intervention

107. No. of arms

Arm 1

108. Intervention received

109. Intervention dosage and regimen

110. How many participants got randomized?

111. No. of males

112. Mean age (years, standar deviation)

Arm 2

Skip if one treatment arm tested this intervention type.

113. Intervention received

114. Intervention dosage and regimen

115. How many participants got randomized?

116. No. of males

117. Mean age (years, standar deviation)

Arm 3

Skip if one treatment arm tested this intervention type.

118. Intervention received

119. Intervention dosage and regimen

120. How many participants got randomized?

121. No. of males

122. Mean age (years, standar deviation)

Arm 4 onwards

Use this section if four or more treatment arms received the intervention type (enter the detail of respective treatment arms in separate lines).

123. Intervention received

124. Intervention dosage and regimen

125. How many participants got randomized?

126. No. of males

127. Mean age (years, standar deviation)

Data of placebo and/or no intervention receiving arm

128. Intervention received

129. Intervention dosage and regimen

130. How many participants got randomized?

131. No. of males

132. Mean age (years, standar deviation)

Outcome data

133. Select (tick) outcomes reported in this study. *

Check all that apply.

	Reported	Not reported
Cesearen section	☐	☐
Pre-eclampsia	☐	☐
Polyhydramnios	☐	☐
Preterm birth	☐	☐
Macrosomia	☐	☐
Prolonged labour	☐	☐
Gestational hypertension	☐	☐
Premature rupture of membranes	☐	☐
Neonatal hypoglycemia	☐	☐
Newborn hyperbilirubinemia	☐	☐
Congenital anomaly	☐	☐
Apgar scores at 1 min	☐	☐
Apgar scores at 5 min	☐	☐
Birth weight	☐	☐
Birth length	☐	☐
Gestational age at birth	☐	☐
Neonatal Corpulence Index	☐	☐

This content is neither created nor endorsed by Google.

Google Forms
