# Supplementary material for: Comparative effectiveness of adjunct non-pharmacological interventions on maternal and neonatal outcomes in gestational diabetes mellitus patients: A systematic review and network meta-analysis protocol of randomized controlled trials
Source: PLoS One. 2022 Jan 27;17(1):e0263336. doi: 10.1371/journal.pone.0263336 (PMC8794170; doi:10.1371/journal.pone.0263336)
Supplement: S3 File — (PDF) [file pone.0263336.s004.pdf]

Data abstraction form for analysis

Title: Comparative effectiveness of adjunct non-pharmacological interventions on maternal and neonatal outcomes in gestational diabetes mellitus patients: A systematic review and network meta-analysis protocol of randomized controlled trials.

Draft copy

Author: Dr. Sumanta Saha

Dichotomous outcomes

First author’s last name	Year of publication	Title of the study	Outcome	Intervention given in the treatment arm	Events	Total participants (included in the analysis)	DOI	PMID

Continuous outcomes

First author's last name	Year of publication	Title of the study	Outco me	Intervention given in the treatment arm	Unit (as reported in the trial; no conversion)	Mean	Standard deviation	Total participants (included in the analysis)	DOI	PMID
