# Supplementary material for: Comparative effectiveness of adjunct non-pharmacological interventions on maternal and neonatal outcomes in gestational diabetes mellitus patients: A systematic review and network meta-analysis protocol of randomized controlled trials
Source: PLoS One. 2022 Jan 27;17(1):e0263336. doi: 10.1371/journal.pone.0263336 (PMC8794170; doi:10.1371/journal.pone.0263336)
Supplement: Attachment — Submitted filename: RESPONSE TO REVIEWERS.docx [file pone.0263336.s005.docx]

**RESPONSE TO REVIEWERS’ COMMENTS**

Dear Reviewers,

Thank you for reading the manuscript critically and sharing your insightful comments and suggestions. The manuscript has been thoroughly revised based on your advice. Additionally, several sentences have been edited to ensure a concise and logical presentation. Two manuscript copies, one with track changes enabled and another clean version with changes in the colored text, are also submitted. Below (in red colored text) are the point-wise responses to your comments.

1. Does the manuscript provide a valid rationale for the proposed study, with clearly identified and justified research questions?

The research question outlined is expected to address a valid academic problem or topic and contribute to the base of knowledge in the field.

Reviewer #1: Yes

**REPLY:**

Thank you for your feedback.

Reviewer #2: Partly

**REPLY:**

Thank you for your comment. The rationale has been reinforced further based on your other comments below. A clarification has been added now to the ‘Introduction’ section (the last two paras particularly).

2. Is the protocol technically sound and planned in a manner that will lead to a meaningful outcome and allow testing the stated hypotheses?

The manuscript should describe the methods in sufficient detail to prevent undisclosed flexibility in the experimental procedure or analysis pipeline, including sufficient outcome-neutral conditions (e.g. necessary controls, absence of floor or ceiling effects) to test the proposed hypotheses and a statistical power analysis where applicable. As there may be aspects of the methodology and analysis which can only be refined once the work is undertaken, authors should outline potential assumptions and explicitly describe what aspects of the proposed analyses, if any, are exploratory.

Reviewer #1: Yes

**REPLY:**

Thank you for your feedback.

Reviewer #2: Partly

**REPLY:**

Thank you for your comment. The methodology has been elaborated further based on your comments below. Detailed responses are below (at ‘6. Review Comments to the Author’).

3. Is the methodology feasible and described in sufficient detail to allow the work to be replicable?

Descriptions of methods and materials in the protocol should be reported in sufficient detail for another researcher to reproduce all experiments and analyses. The protocol should describe the appropriate controls, sample size calculations, and replication needed to ensure that the data are robust and reproducible.

Reviewer #1: No

**REPLY:**

Thank you for your comment. The analysis plan has been clarified further based on comments made by both reviewers. Please also see replies below about the amendments made in eligibility criteria and analysis (at ‘6. Review Comments to the Author’).

Reviewer #2: Yes

**REPLY:**

Thank you for your feedback.

4. Have the authors described where all data underlying the findings will be made available when the study is complete?

The PLOS Data policy requires authors to make all data underlying the findings described in their manuscript fully available without restriction, with rare exception, at the time of publication. The data should be provided as part of the manuscript or its supporting information, or deposited to a public repository. For example, in addition to summary statistics, the data points behind means, medians and variance measures should be available. If there are restrictions on publicly sharing data—e.g. participant privacy or use of data from a third party—those must be specified.

Reviewer #1: No

**REPLY:**

A data availability statement is now included in the manuscript just before the references.

Reviewer #2: No

**REPLY:**

A data availability statement is now included in the manuscript just before the references.

5. Is the manuscript presented in an intelligible fashion and written in standard English?

PLOS ONE does not copyedit accepted manuscripts, so the language in submitted articles must be clear, correct, and unambiguous. Any typographical or grammatical errors should be corrected at revision, so please note any specific errors here.

Reviewer #1: Yes

**REPLY:**

Thank you for your feedback.

Reviewer #2: No

**REPLY:**

The manuscript has been hard edited meticulously. Besides responding to the reviewers' comments, every sentence was re-read and amended when required to ensure lexical integrity.

6. Review Comments to the Author

**Reviewer #1:** In general this is a well designed systematic review / meta-analysis, that is already registered on PROSPERO. A good deal of thought has gone into the searches and terms used. I think it will make a useful contribution to the literature.

**REPLY:**

Thank you for finding the proposed review relevant.

A couple of minor issues.

I would use the updated PRISMA guidelines and Checklist, rather than the 2015 version.

**REPLY:**

Thanks for the comment. By 'updated PRISMA guideline,' if you are referring to the PRISMA-2020 guideline for systematic review and meta-analysis, please allow me to clarify that it will not apply to this manuscript since this is a protocol for a systematic review and not the review itself.

Nevertheless, based on your comment, I searched the PRISMA website for any updated version of the PRISMA-P statement. However, I couldn't find any updated version of PRISMA-P after 2015. Additionally, I contacted one of the authors of the PRISMA 2020 statement for the same, and I learned that the PRISMA-P 2015 version is the only version available for systematic review protocols presently.

A humble request, if you are referring to any other version of the PRISMA statement, kindly let me know.

PMA isn't defined for the reader, presumably it is primary meta-analysis?

**REPLY:**

I apologize for not mentioning the full form of the PMA. PMA stands for pairwise meta-analysis. The abbreviation's use has been updated accordingly in the manuscript.

It isn't entirely clear what the effect sizes will be in the meta-analysis, and which formulas will be used to compute these effect sizes and so on.

**REPLY:**

Thank you for the comments. These issues are now addressed in the manuscript. I quote the sentence for your kind reference- 'The ES estimation of categorical and continuous outcomes will ensue in the risk ratio and weighted or standardized mean difference, respectively, for both NMA and PMA.' NMA stands for network meta-analysis.

Regarding formulae, these are specified in the updated manuscript in the ‘Methods’ section under the subheading ‘Criteria for selecting outcomes for NMA.’

Are the authors planning to look at other measures of bias, e.g. small study / publication bias? If so, what techniques will be used.

**REPLY:**

Yes, publication bias assessment will happen. However, the description of it was incomplete in the manuscript. Thanks for pointing it out. Publication bias assessment will ensue using comparison-adjusted funnel plots, and this is now mentioned in the manuscript in the ‘Methods’ section (under subheading ‘RoB across studies’).

**Reviewer #2:** Major comments:

The use of non-pharmacologic lifestyle intervention (specifically, diet and exercise) and monitoring of blood glucose levels for the initial management of gestational diabetes (GDM) is standard of care. The determination of which of these accepted interventions has a more pronounced impact on pre-specific maternal and neonatal outcomes among women with GDM and their offspring would be of interest. However, the authors do not describe a study to compare effectiveness of currently accepted interventions for the management of GDM, but instead describe a comparison of non-pharmacologic interventions that are not adopted by current international guidelines due to a lack of evidence for benefit.

Thus, the hypothesis and goals of the review are not clear or consistent in the abstract or introduction of this manuscript.

**REPLY:**

Thank you for your comment. A modified rationale of the manuscript has been drafted (please see the last two paragraphs of the introduction section for these amendments). The manuscript now coins on the weaknesses of the existing trials and meta-analyses of these trials to reinforce its justification. Furthermore, to make the abstract and introduction consistent, the interventions and outcomes of interest are stated explicitly in both sections.

Furthermore, the validity of comparing across interventions such as omega-3 supplementation or smartphone applications, which prior studies did not show to differ from placebo, and why a network meta-analysis of these interventions should be performed, is questionable. Finally, the manuscript is not fully intelligible and some aspects are difficult to follow.

**REPLY:**

Thanks for raising the issue. Your comment makes sense; a comparison between omega-3 supplementation versus smartphone application in terms of efficacy is perhaps two distant entities to make a meaningful comparison. Based on your comment, the analysis plan got updated to a more rationalized form. To describe using the above example, instead of comparing omega-3 supplementation versus smartphone, now juxtaposition will happen between the parent categories of these interventions (i.e., nutritional supplementation versus digital application).

The updated analysis plan will compare the following interventions- nutritional supplementation, digital intervention, structured exercise program, educational program, counseling service, or a combination of these.

An additional network meta-analysis will separately compare various nutritional supplements to address your concern in the subsequent comment. It will help understanding how supplements like vitamin D, probiotics, omega-3 fatty acids, etc., differ in terms of the occurrence of the perinatal outcomes in GDM patients and their neonates.

Please find this updated plan introduced briefly in the 'Introduction' section and described elaborately under the 'Methods' section (beneath the 'NMA' sub-heading).

Minor comments:

-Inclusion criteria must be more specific: the types of interventions that will be included must be described and specified (eg., vitamin D, omega-3, low carb diet, etc. etc.); the specific outcomes that will be evaluated must be described and specified

**REPLY:**

Thank you for the advice. The inclusion criteria have got updated now.

Regarding interventions', a list of interventions of interest has been specified (as stated in response to your previous comment). Also, following your suggestion, particular nutritional interventions will get compared in the additional network meta-analysis models (as specified in response to your previous comment).

However, an explicit list of nutritional interventions (like vitamin D, probiotics, omega-3 fatty acids, etc.) to be studied in the additional network meta-analysis models isn't prepared to keep the scope of the review broad. It will plausibly enhance the comprehensiveness (by allowing all possible nutritional supplement types tested in clinical trials on GDM patients to get included in the review) and rigor of the evidence.

Next, the reason for not including interventions like a low carbohydrate diet and self-monitoring of blood glucose in the proposed review is that these are part of standard GDM care. Notably, the non-pharmacological adjuncts to standard GDM care are the interventions of interest. In other words, the prospective review aims to study non-pharmacological interventions' roles on perinatal outcomes when combined with standard/usual GDM care.

Concerning outcomes, now specific outcomes of interest have been enlisted under the inclusion criteria as per your suggestion.

-How many authors will perform the review specifically? (“3 or more”?)

**REPLY:**

Three authors will perform the review.

-Page 10: “Both pharmacological and non-pharmacological interventions will not get included in the same NMA model.” – what pharmacologic interventions are referred to here, as the review purports to compare non-pharmacologic interventions?

**REPLY:**

Thanks for identifying the typing error. The sentence has been removed from the revised manuscript.

Thank you.
