# Supplementary material for: Comparative effectiveness of adjunct non-pharmacological interventions on maternal and neonatal outcomes in gestational diabetes mellitus patients: A systematic review and network meta-analysis protocol of randomized controlled trials
Source: PLoS One. 2022 Jan 27;17(1):e0263336. doi: 10.1371/journal.pone.0263336 (PMC8794170; doi:10.1371/journal.pone.0263336)
Supplement: Attachment — Submitted filename: R2_RESPONSE TO REVIEWERS.docx [file pone.0263336.s006.docx]

Dear Reviewers,

I sincerely thank you for reading the manuscript and sharing your insightful comments. Please find two copies of the manuscript attached- one with track changes and the other a clean version.

The manuscript file accompanies four supporting documents-

1.	S1 Table (relevancy wise search strings)

2.	S1 File (PRISMA-P checklist)

3.	S2 File (data abstraction form)

4.	S3 File (data abstraction form for analysis)

Please find the responses to your comments below.

Review Comments to the Author

Reviewer #3: The authors provided us with a protocol for a systematic review and network meta-analysis for non-pharmacological interventions in gestational diabetes mellitus. The protocol is well structured and clear to read. Nevertheless, a variety of issues need to be covered before considered for publication.

1. Outcomes: It would be preferable to create an exhaustive list of outcomes based on the literature to ensure maximum objectiveness in the conducting of the review. This is not provided by the authors. Few examples are given e.g. preeclampsia. Still, it is to our belief necessary to prespecify (in the best possible way) outcome measures to be studied.

**Author’s reply:**

Thank you for the feedback. The updated version of this manuscript contains the following 17 outcomes as stated under the ‘Methods’ section in the manuscript –

1. Cesarean section
2. Pre-eclampsia
3. Polyhydramnios
4. Preterm birth
5. Macrosomia
6. Prolonged labor
7. Gestational hypertension
8. Premature rupture of membranes
9. Neonatal hypoglycemia
10. Neonatal hyperbilirubinemia
11. Congenital anomaly
12. Apgar scores at 1 min
13. Apgar scores at 5 min
14. Birth weight
15. Birth length
16. Gestational age at birth
17. Neonatal Corpulence Index

2. Details on reports providing information on more than one outcome have not been given.

**Author’s reply:**

Thank you for the comment. Now the following statement has been included in the revised manuscript to address the issue- “When multiple outcomes' data are reported in an article, it will get captured in the S2 and S3 Files and subsequently reported in the summary table of the proposed review.”

3. Study selection: very weakly presented part. There is no specification of the program to be used or of the strategy (two separate authors etc). This needs to be analysed.

**Author’s reply:**

Thank you for the comment. The study selection section has been revised, and the updated version is quoted here for your kind reference- “The retrieved citations will get uploaded in the Rayyan systematic reviews software for study selection.[18] After eliminating the duplicate articles, two review authors will independently skim through the titles and abstracts of the remaining and shortlist the seemingly eligible and dubious ones for full-text reading.” Kindly note, as the subheading “Role of review authors” states how a conflict in an opinion between the review authors will get resolved, it's not restated here.

4. RoB evaluation using the 2011 tool needs to be justified. To our knowledge the tool has already been updated. Why is the newest form not chosen?

**Author’s reply:**

Thank you for the comment. The updated version of the Cochrane RoB tool will be used. It’s quoted here from the revised manuscript for your kind reference- “The Revised Cochrane risk-of-bias tool for randomized trials (RoB 2) will be used for the RoB assessment of the respective studies.[19] Utilizing signaling questions, the RoB will get assessed in the following bias domains- randomization process, intended interventions, missing outcome data, outcome data measurement, and reported results. Each of the signaling questions can have any of the following answers based on the review authors' judgment- yes, probably yes, probably no, no, and no information. For each domain, an assessment of the algorithm-based evaluation of the signaling questions will transpire to incorporate necessary changes. The overall RoB judgment will depend on the evaluations made for respective domains (detailed elsewhere).[19] Two review authors will independently pursue the RoB assessment.” Kindly note, as the subheading “Role of review authors” states how a conflict in an opinion between the review authors will get resolved, it's not restated here.

5. Prespecified forms of data extraction are not given. They are only described. Please provide a pdf version of the form.

**Author’s reply:**

Thank you for the comment. Please find a draft data abstraction form attached to the revised manuscript. Besides, a draft data collection sheet for capturing data for meta-analysis is also included. Kindly refer to the attached S2 File and S3 File.

6. Issues of the network meta-analysis are correct but are not backed up with bibliography. For example handling of zero events.

**Author’s reply:**

Thank you for the feedback. Following additional relevant citations to back up the network meta-analysis are now included in the revised manuscript-

1. White IR. Network Meta-analysis. Stata J Promot Commun Stat Stata [Internet]. 2015;15:951–85. Available from: http://journals.sagepub.com/doi/10.1177/1536867X1501500403

2. Salanti G, Ades AE, Ioannidis JPA. Graphical methods and numerical summaries for presenting results from multiple-treatment meta-analysis: an overview and tutorial. J Clin Epidemiol [Internet]. 2011;64:163–71. Available from: https://linkinghub.elsevier.com/retrieve/pii/S0895435610001691

3. Rouse B, Chaimani A, Li T. Network meta-analysis: an introduction for clinicians. Intern Emerg Med [Internet]. 2017;12:103–11. Available from: http://link.springer.com/10.1007/s11739-016-1583-7

7. The biggest issue of the protocol is the search strategy. Even though information on the supplementary material is used to justify the choice of the protocol, we strongly disagree with the assumption of an RCT without adequate expansion of the search strategy. An in depth explanation is in order.

**Author’s reply:**

Thank you for the advice. An expanded search string is now included in the revised manuscript. For your kind reference, I am pasting it here from the revised manuscript- ‘(("nutrition*"[All Fields] OR ("vitamin s"[All Fields] OR "vitamine"[All Fields] OR "vitamines"[All Fields] OR "vitamins"[Pharmacological Action] OR "vitamins"[MeSH Terms] OR "vitamins"[All Fields] OR "vitamin"[All Fields]) OR "probitic"[All Fields] OR ("synbiotics"[MeSH Terms] OR "synbiotics"[All Fields] OR "synbiotic"[All Fields]) OR ("prebiotically"[All Fields] OR "prebiotics"[MeSH Terms] OR "prebiotics"[All Fields] OR "prebiotic"[All Fields]) OR ("calcification, physiologic"[MeSH Terms] OR ("calcification"[All Fields] AND "physiologic"[All Fields]) OR "physiologic calcification"[All Fields] OR "mineralization"[All Fields] OR "mineral s"[All Fields] OR "mineralisable"[All Fields] OR "mineralisation"[All Fields] OR "mineralisations"[All Fields] OR "mineralise"[All Fields] OR "mineralised"[All Fields] OR "mineralising"[All Fields] OR "mineralizations"[All Fields] OR "mineralize"[All Fields] OR "mineralized"[All Fields] OR "mineralizer"[All Fields] OR "mineralizers"[All Fields] OR "mineralizes"[All Fields] OR "mineralizing"[All Fields] OR "minerals"[MeSH Terms] OR "minerals"[All Fields] OR "mineral"[All Fields]) OR ("digital"[All Fields] OR "digitalisation"[All Fields] OR "digitalised"[All Fields] OR "digitalization"[All Fields] OR "digitalize"[All Fields] OR "digitalized"[All Fields] OR "digitalizer"[All Fields] OR "digitalizing"[All Fields] OR "digitally"[All Fields] OR "digitals"[All Fields] OR "digitization"[All Fields] OR "digitizations"[All Fields] OR "digitize"[All Fields] OR "digitized"[All Fields] OR "digitizer"[All Fields] OR "digitizers"[All Fields] OR "digitizes"[All Fields] OR "digitizing"[All Fields]) OR ("smartphone"[MeSH Terms] OR "smartphone"[All Fields] OR "smartphones"[All Fields] OR "smartphone s"[All Fields]) OR ("exercise"[MeSH Terms] OR "exercise"[All Fields] OR "exercises"[All Fields] OR "exercise therapy"[MeSH Terms] OR ("exercise"[All Fields] AND "therapy"[All Fields]) OR "exercise therapy"[All Fields] OR "exercise s"[All Fields] OR "exercised"[All Fields] OR "exerciser"[All Fields] OR "exercisers"[All Fields] OR "exercising"[All Fields]) OR ("educability"[All Fields] OR "educable"[All Fields] OR "educates"[All Fields] OR "education"[MeSH Subheading] OR "education"[All Fields] OR "educational status"[MeSH Terms] OR ("educational"[All Fields] AND "status"[All Fields]) OR "educational status"[All Fields] OR "education"[MeSH Terms] OR "education s"[All Fields] OR "educational"[All Fields] OR "educative"[All Fields] OR "educator"[All Fields] OR "educator s"[All Fields] OR "educators"[All Fields] OR "teaching"[MeSH Terms] OR "teaching"[All Fields] OR "educate"[All Fields] OR "educated"[All Fields] OR "educating"[All Fields] OR "educations"[All Fields]) OR ("counsel"[All Fields] OR "counseled"[All Fields] OR "counselings"[All Fields] OR "counselled"[All Fields] OR "counselling"[All Fields] OR "counseling"[MeSH Terms] OR "counseling"[All Fields] OR "counsellings"[All Fields] OR "counsels"[All Fields])) AND (("diabetes, gestational"[MeSH Terms] OR "gestational diabetes mellitus"[Title/Abstract] OR "GDM"[Title/Abstract]) AND "randomized controlled trial"[Publication Type] AND "randomized controlled trial"[Publication Type])) AND (randomizedcontrolledtrial[Filter]).’

Thank you.

Reviewer #4: The present protocol is well designed and presented.

My comments are the following.

COMMENT 1. Only manuscripts in english are considered eligible for inclusion, please include more languages.

**Author’s reply:**

Thank you for your comment. The review authors are adept in the English language only. Moreover, no funding is available for hiring language translators. It is a potential limitation of the proposed review, now mentioned in the revised manuscript under the subheading ‘Limitation’ using the following text- “As review authors of the proposed review are adept in the English language only, articles published in any other language will not get included in this review.”

COMMENT 2. Please extend the search strategy to sources of grey literature.

**Author’s reply:**

Thank you for the comment. The search strategy has now been extended to a pre-print server medRxiv to include non-peer-reviewed articles. It has been stated in the revised manuscript using the following sentence- “Additional searches will ensue in the bibliography of articles recruited in this review and a preprint server, medRxiv.[17]”

Thank you.
